# The Effect of Antiretroviral Therapy on SIRT1, SIRT3 and SIRT6 Expression in HIV-Infected Patients

**DOI:** 10.3390/molecules27041358

**Published:** 2022-02-17

**Authors:** Karolina Jurkowska, Beata Szymańska, Brygida Knysz, Agnieszka Piwowar

**Affiliations:** 1 Department of Toxicology, Faculty of Pharmacy, Wroclaw Medical University, Wybrzeże L. Pasteura 1, 50-367 Wroclaw, Poland; karolina.jurkowska@student.umw.edu.pl (K.J.); agnieszka.piwowar@umw.edu.pl (A.P.); 2 Department of Infectious Diseases, Liver Diseases and Acquired Immune Deficiencies, Faculty of Medicine, Wroclaw Medical University, Wybrzeże L. Pasteura 1, 50-367 Wroclaw, Poland; brygida.knysz@umw.edu.pl

**Keywords:** SIRT1, SIRT3, SIRT6, cART, HIV, comorbidities, sirtuins

## Abstract

Human Immunodeficiency Virus (HIV) infection and the chronic use of combined antiretroviral therapy (cART) may affect the occurrence of certain disturbances in the body. There is growing interest in sirtuins–enzymes involved in the regulation of many metabolic processes in the organism and in the pathogenesis of many diseases which also exhibit potential antiviral activity. The aim of the study was to investigate the connection of cART to the expression of Sirtuin 1 (SIRT1), Sirtuin 3 (SIRT3) and Sirtuin 6 (SIRT6) in HIV-infected men. The plasma levels of sirtuins were measured before and one year after cART, and related to HIV viral load, lymphocytes T CD4+ and CD8+ count as well as the applied cART. The levels of sirtuins in plasma were measured in HIV-infected patients (*n* = 53) and the control group (*n* = 35) by immunoassay methods. There were statistically significant (*p* < 0.05) differences between SIRT6 in the HIV-infected patients before therapy and in the subgroups, depending on the count of lymphocytes T CD8+. There were significant differences in the levels of SIRT1 depending on the applied treatment regimen. The obtained results indicate the most significant changes in the expression of SIRT6 in the course of HIV infection and suggest an influence of the type of cART on the level of SIRT1, which indicates its important role in the course of HIV.

## 1. Introduction

About 37.9 million people worldwide are currently infected with the Human Immunodeficiency Virus (HIV) [1]. Due to the introduction of Highly Active Antiretroviral Therapy (HAART), the life expectancy of people infected with HIV comes close to that of the general population [2]. Despite the use of an effective method of treatment–combined antiretroviral therapy (cART)–and the resulting significant reduction in mortality from Acquired Immunodeficiency Syndrome (AIDS), it is still a serious socioeconomic and health problem [3,4].

The introduction of cART has proved to be a breakthrough in the treatment of HIV infection. This therapy involves the use of at least three drugs from different available pharmacological groups, ensuring the inhibition of viral replication to levels undetectable by the most sensitive analytical methods; it prevents the development of drug resistance and enables the restoration of immune system function as well as preventing or delaying the occurrence of AIDS [5]. There are different therapeutic regimens based on the application of at least two nucleoside reverse transcriptase inhibitors (NRTIs) in combination with non-nucleoside reverse transcriptase inhibitors (NNRTIs), integrase transfer inhibitors (INSTIs), protease inhibitors (PIs), fusion inhibitors and C-C Chemokine Receptor 5 (CCR5) antagonists. In addition to two NRTIs, recommended regimens include protease inhibitors (PIs) or integrase transfer inhibitors (INSTIs) [6,7,8].

PIs act at a late stage in the replication cycle of the virus, inhibiting the activity of the protease enzyme, which in turn prevents the breakdown of structural and enzymatic protein precursors of the gag and gag-pol virus proteins, leading to the formation of immature, non-infectious virions, unable to initiate another replication cycle. PIs are characterized by a low risk of drug resistance (high genetic barrier) and high effectiveness [5]. Almost all PIs are inhibitors of the Cytochrome P450 3A4 (CYP3A4) isoenzyme, resulting in a high risk of drug interactions. Ritonavir is a particularly potent inhibitor of CYP3A4, which is used to potentiate the effects of the remaining PIs (booster), allowing for a simplified dosing schedule and lower toxicity. Cobicistat is also used to boost PIs [9]. However, PIs are burdened with numerous side effects, mainly related to disorders of adipose tissue and glucose metabolism (dyslipidemia, impaired glucose tolerance, insulin resistance etc.) [10].

INSTIs are a new class of antiretroviral drugs with a high safety profile and efficacy. The first INSTI was raltegravir, registered by the Food and Drug Administration (FDA) in 2007 [5]. INSTIs block the integrase enzyme, which catalyzes the formation of covalent bonds between the viral and host DNA, preventing the incorporation of viral DNA into the host genome. Compared to PIs, they are burdened with a lower risk of metabolic disorders; however, they are characterized by a lower genetic barrier (except dolutegravir and bictegravir) [11]. 

Since the approval of the first antiretroviral drug zidovudine in 1987, significant progress has been made in the treatment of HIV infection. Currently, more than 20 different active substances are available. Older drugs are gradually being replaced by new, much less toxic derivatives. For example, the currently used NNRTIs abacavir, tenofovir disoproxil fumarate, and tenofovir alafenamide, compared to the old generation zidovudine and didanosine, are less frequently associated with the occurrence of serious side effects such as mitochondrial toxicity, lipodystrophy, lipoatrophy, hepatotoxicity and hematological disorders [7,9]. Clinical trials on the use of a regimen other than cART are also being conducted in order to maintain high effectiveness and minimize the risk of treatment complications [12]. The first (since 2017) two-drug therapy was a combination of dolutegravir and rilpivirine [13,14]. Another new preparation is a combination of dolutegravir and lamivudine [15].

At present, patient care in terms of comorbidities, especially those associated with accelerated aging, long-term therapy and a chronic inflammation state, are equally important as effective antiretroviral treatment. The quick identification of comorbidities and the implementation of appropriate prophylactic or therapeutic procedures seems particularly crucial [1,2]. In recent years, there have been many reports on enzymes from the sirtuin family that have indicated their participation in the modulation of many metabolic processes [16,17]. Sirtuins (SIRT 1–7) are evolutionarily conserved, NAD+ dependent class III deacetylases which regulate gene expression mainly by deacetylation of histones and other enzymatic, structural or transcription factors [18]. They are also characterized by other enzymatic activities: ADP ribosylation (SIRT1, SIRT4 and SIRT6), desuccinylation and demalonylation (SIRT5), delipoylation (SIRT4), demyristoylation and depalmitoylation (SIRT6), and they have different cell localization: nuclear (SIRT1, SIRT6, SIRT7), cytoplasmic (SIRT2), and mitochondrial (SIRT3, SIRT4, SIRT5). So far, in terms of the development and course of many diseases, the best known are SIRT1, SIRT3, and SIRT6 [19]. Numerous studies on the use of activators or inhibitors of sirtuin activity in the treatment of neoplastic or metabolic diseases, mainly type 2 diabetes (T2DM), are currently being conducted [20,21]. Data on sirtuins in HIV-infected individuals are rare and mainly relate to SIRT1 [22,23]. To the best of the authors′ knowledge, there are no data available on other sirtuins in the course of HIV infection.

SIRT1 expression has been demonstrated in most tissues, including skeletal muscles, liver, adipocytes, kidneys etc. SIRT1 has been shown to participate in multiple signaling pathways related to gluconeogenesis, glycolysis, insulin secretion, DNA repair, aging, and lipid metabolism [16]. It regulates the activity of many transcription factors, including forkhead box transcription factors (FOXOs), hypoxia-inducible factor 1-alpha (HIF-1α), liver X receptor (LXR), sterol regulatory element-binding protein 1 (SREBP-1), peroxisome proliferator-activated receptor gamma co-activator 1alpha (PGC-1α), p53, and nuclear factor kappa-light-chain-enhancer of activated B cells (NFkB) [22]. The interaction of SIRT1 with the HIV viral protein Trans-Activator of Transcription (Tat), which is a transcriptional Trans activator of integrated proviral mRNA, has also been described [24]. Deacetylation of Tat increases the efficiency of HIV transcription by continuing the elongation of viral mRNA. Tat also blocks SIRT1, thereby reducing NFkB deacetylation, leading to the activation of inflammatory processes. SIRT1 may have a significant and direct impact on the process of HIV infection and chronic immune activation–one of the causes of accompanying comorbidities [23,24].

SIRT3 is the major mitochondrial deacetylase responsible for maintaining proper ATP levels in cells. It regulates the activity of pyruvate dehydrogenase (PDH) and is responsible for the maintenance of energy homeostasis in skeletal muscles [25]. In the liver, it also regulates the processes of beta fatty acid oxidation through long-chain acyl-CoA dehydrogenase (LCAD) deacetylation. In addition, SIRT3 increases the expression of PGC-1α and the uncoupling of Protein 1 (UCP1) in brown adipose tissue (BAT), thus enhancing thermogenesis under the influence of calorie restriction. SIRT3 also affects antioxidant processes through deacetylation and increasing the activity of the antioxidant enzymes superoxide dismutase 2 (SOD2) and catalase (CAT) in a forkhead box protein O3a (FOXO3a) dependent manner [26]. 

SIRT6, located in the cell nucleus, mainly catalyzes the reactions of mono- ADP-ribosylation and the deacetylation of histone 9 proteins (H3K9ac, H3K56ac and H3K18ac) as well as transcriptional factors responsible for aging processes, metabolism and inflammatory processes, e.g., NF-κB, hypoxia-inducible factor 1 (HIF-1) and cellular transcription factor (c-Myc) [27]. SIRT6 is a critical regulator of DNA repair in telomeric regions and a positive regulator of longevity through its influence on the metabolism and telomere functions [28]. SIRT6 participates in the processes of DNA repair in various mechanisms, including the up-regulation of a double-strand break (DSB) repair factor–DNA-dependent protein kinase (DNA-PK) and SNF2H, a chromatin-remodeling factor, ensuring genome stability and acting as a tumor suppressor [16]. SIRT6 regulates the process of glycolysis by deacetylation of HIF-1α and, consequently, reducing the expression of glycolytic genes, including Glucose transporter-1 (GLUT1), lactate dehydrogenase (LDH), phosphofructokinase-1 (PFK1) and pyruvate dehydrogenasekinase-1 (PDK1). [18,29] It also influences the process of gluconeogenesis through deacetylation of PGC-1α, increasing the expression of gluconeogenic genes as a result. However, SIRT6 also regulates the expression of gluconeogenesis in a FOXO1-dependent mechanism by reducing the expression of these genes. Moreover, SIRT6 is a positive regulator of beta oxidation of fatty acids in the liver through the deacetylation of Lys780 of Nuclear Receptor Coactivator 2 (NCOA2) and the activation of Peroxisome proliferator-activated receptor alpha (PPARα) [16,27]. By deacetylating H3K9 in the NFkB promoter region and Lys310 of the p65 subunit (RelA) of NFkB, SIRT6 inhibits proinflammatory activity [28].

Detailed data on the mechanisms of the development of co-morbidities resulting from HIV infection as well as detailed data on the effects of cART are still limited. Due to the various functions performed by sirtuins in the regulation of many physiological processes, abundant data indicating their participation in the pathogenesis of different diseases, growing scientific interest in the role of these enzymes, and the lack of, or insufficient data on, the role of selected sirtuins–SIRT1, SIRT3 and SIRT6–in the course of HIV infection, the aim of the study was to show possible changes in the expression of these selected sirtuins during antiretroviral therapy. The plasma levels of SIRT1, SIRT3, and SIRT6 were measured before and one year after cART, and related to HIV viral load, lymphocytes T CD4+ and CD8+ count, and the applied treatment regimen. The obtained data will allow for a preliminary assessment of the influence of selected sirtuins on the course of HIV infection and cART therapy.

## 2. Results

The study group consisted of HIV-infected men before cART (group A) and one year after the implementation of cART (group B, HIV-infected men treated with the two therapeutic regimens: INSTIs or PIs), and the control group (group C) consisted of non-HIV-infected men. 

All demographic and clinical data of the study and control group are presented in Table 1, and immunological data concerning the patient group are provided in Table 2.

There were no statistically significant differences (*p* > 0.05) in age, BMI (Body Mass Index), or values of basic biochemical parameters such as TC (total cholesterol), LDL (low-density lipoprotein), HDL (high-density lipoprotein), TG (triglycerides) and FBG (fasting blood glucose) between groups A, B and C. The differences between median HIV viral load, lymphocytes T (LT) CD4+ and LT CD8+ count in patients before and after treatment were statistically significant. 

Median levels, interquartile ranges and statistical analysis for SIRT1, SIRT3 and SIRT6 in the pre-treatment (A) and post-treatment (B) of HIV-infected men and the control group (C) are provided in Table 3.

The median level of SIRT1 in the group of HIV-infected men before treatment (A) was over 1.5-fold higher compared to the group after treatment (B), and almost 1.2-fold lower than in the control group (C). The median level of SIRT1 in the group after cART was 1.8-fold lower compared to the median level of SIRT1 in the control group. However, these differences were not statistically significant. Median levels of SIRT3 were similar in both groups of HIV-infected men (A and B) and were approximately 1.4-fold lower compared to the control group but without statistical significance. There were statistically significant (*p* < 0.05) differences in plasma levels of SIRT6 in pre-treatment (A), post-treatment (B) and control (C) groups (*p* = 0.003). Post hoc analysis showed a statistically significant difference in the SIRT6 plasma level in the pre-treatment group (A) compared to the post-treatment group (B) (almost 1.6-fold lower; *p* = 0.022) and statistically significant differences between the median level of SIRT6 in the pre-treatment group (A) compared to the control group (almost 2.6-fold lower; *p* = 0.007), (Table 2). 

The median levels and interquartile ranges with statistical analysis of examined sirtuins in the plasma of HIV-infected men before cART (A) and after cART (B) are presented in Table 4 and Table S1 and divided into subgroups according to LT CD4+ count ≤300 cells/μL or >300 cells/μL. 

In group A, before cART, 18 (34%) patients had an LT CD4+ count ≤ 300 cells/μL and 35 (66%) an LT CD4+ count > 300 cells/μL. In the group after cART (B), 10 (19%) patients had an LT CD4+ count ≤ 300 cells/μL and 43 (81%) an LT CD4+ count > 300 cells/μL. There was a downward trend in SIRT1, SIRT3 and SIRT6 levels with an increase in LT CD4+ count >300 cells/μL by 53%, 66% and 53%, respectively, in the pre-treatment group (A), and by 32%, 16% and 24% in the post-cART group (B). There was no statistically significant difference between SIRT1, SIRT3 and SIRT6 levels in subgroups with LT CD4+ count ≤300 cells /µL before cART therapy and the levels of those sirtuins in the subgroup with LT CD4+ count ≤ 300 cells /µL after treatment. Similar results were obtained in subgroups with LT CD4+ count > 300 cells /µL before and after treatment (Table 4).

The median levels and interquartile ranges with statistical analysis of examined sirtuins in the plasma of HIV-infected men before cART (A) and after cART (B), divided into subgroups according to LT CD8+ count ≤ 1000 cells/μL and >1000 cells/μL, are presented in Table 5 and Table S2.

In group A, before cART, 26 (49%) HIV-infected men had an LT CD8+ count ≤1000 cells/μL and 27 (51%) an LT CD8+ count > 1000 cells/μL. In the group after cART (B), 36 (68%) patients had an LT CD8+ count ≤ 1000 cells/μL and 17 (32%) an LT CD8+ count > 1000 cells/μL. 

There was a downward trend in the level of SIRT1 and SIRT3 with an increase in LT CD8 + count > 1000 cells/μL by 56% and 47%, respectively, in the pre-cART group (A) and by 39% and 61% in the post-cART group (B). A statistically significant difference was demonstrated between SIRT6 levels in the subgroup with LT CD8+ count ≤ 1000 cells/µL before cART and SIRT6 in the subgroup with LT CD8+ count ≤ 1000 cells/µL after treatment (*p* = 0.04). A statistically significant difference was also demonstrated between SIRT6 levels in the subgroup with LT CD8+ count > 1000 cells/µL before cART treatment and SIRT6 in the subgroup with LT CD8+ count > 1000 cells/µL after treatment (*p* = 0.01). Such a relationship was not detected for SIRT1 and SIRT3 (Table 5).

Due to low HIV viral load after antiretroviral treatment (mean 20 copies/mL in group B), sirtuins in the subgroups with HIV RNA ≤100,000 copies/mL and HIV RNA >100,000 copies/mL were compared only in HIV-infected men prior to cART (A), as shown in Table 6.

In group A, before cART, 22 (42%) of HIV-infected men had ≤100,000 HIV RNA copies/mL and 31 (58%) had >100,000 copies/mL. It was not possible to create subgroups depending on HIV RNA ≤ 100,000 copies/mL and HIV RNA > 100,000 copies/mL in group B, because all patients in this group had a viral load below 100,000 copies/mL. Therefore, the sirtuin levels for this group coincide with the data shown in Table 3. There were no statistically significant differences between the levels of SIRT1 and SIRT3 in the subgroup of HIV-infected men with HIV RNA ≤ 100,000 copies/mL and in the HIV-infected men with HIV RNA > 100,000 copies/mL in the pre-treatment group (A). There was an upward trend in SIRT1 and SIRT3 levels with an increase in HIV RNA >100,000 copies/mL by 41% and 34%, respectively. The median level for SIRT6 in the subgroup with HIV RNA ≤ 100,000 copies/mL was twofold lower than the median level in the subgroup with >100,000 HIV RNA copies/mL, but the difference was not statistically significant. 

Medians and interquartile ranges for SIRT1, SIRT3 and SIRT6 in the plasma of HIV-infected men treated with Protease inhibitors (PIs) and Integrase transfer inhibitors (INSTIs) are presented in Table 7.

Interesting results were obtained in the analysis of sirtuins expression depending on the treatment regimen used–PIs or INSTIs. The median levels of all sirtuins were lower in HIV-infected men treated with PIs compared to the median levels shown in HIV-infected men treated with INSTIs: 4-, 3.3-, and 3.4-fold, respectively. A significant difference between SIRT1 levels was found in the subgroups of HIV-infected men receiving PIs and INSTIs therapy (*p* = 0.025) and not demonstrated for SIRT3 and SIRT6 (Table 7).

## 3. Discussion

Due to the increasing amount of scientific data on the participation of sirtuins in the pathomechanism and course of many diseases, there is a growing interest in the role of these enzymes in viral diseases, including HIV infection [19,30]. Due to their broad spectrum of activity and their regulation of many life processes or metabolic processes in the organism, their participation in viral infections is highly probable [31,32]. Additionally, there is a growing interest in the use of modifiers of sirtuins activity in different types of therapies, including antiviral therapies [17,30].

The authors’ own study showed that, as a result of one-year cART therapy, the level of SIRT1 in HIV-infected men was decreased when compared to SIRT1 levels in HIV-infected men before treatment. The obtained results may suggest a decrease in SIRT1 expression caused by both HIV infection and the antiretroviral therapy used.

Wang 2020 et al. showed increased NF-kB p65 subunit and signal transducer and activator of transcription 3 (STAT3) acetylation in HIV-associated nephropathy (HIVAN) and decreased SIRT1 expression in the glomeruli of mouse and human HIVAN kidneys. The authors also showed that, in the course of HIVAN, the reduction of SIRT1 expression occurs through a mechanism dependent on miRNA-34a [33]. Zhan et al. showed increased miRNA-34a exposure in human vascular endothelial cells (ECs) and arteries isolated from HIV-positive patients treated with antiretroviral therapy (lopinavir and ritonavir) and cART-naïve (pre-treatment) patients. MiRNA-34 expression was significantly elevated by HIV antiretroviral therapy and promoted miRNA-induced senescence of ECs. The authors indicate that p53 protein is the key factor up-regulating miRNA34a and simultaneously acting as a down-regulator of SIRT1, as demonstrated in in vitro studies, where expression of p53 was significantly increased in ECs treated with Tat and lopinavir with ritonavir. The authors also confirmed that miRNA-34a directly affects SIRT1, and its level is reduced by the Tat protein and antiretroviral drugs (lopinavir, ritonavir) in ECs [34].

The authors’ studies have shown that SIRT1 expression is significantly influenced by the cART treatment regimen. SIRT1 levels were higher in HIV-infected men treated with INSTIs compared to HIV-infected men treated with PIs, which may be interesting for further research.

Di Rosa et al. showed that HeLa cells exposed to HIV-based lentivirus and newly synthesized inhibitors of SIRT3 and SIRT2 reduced the rate of viral DNA integration into the host genome to an extent similar to raltegravir. In contrast, exposure to resveratrol significantly increased HIV DNA integration into the host genome. The potential involvement of sirtuins with DNA-fixing proteins such as Ku70 (SIRT3 and SIRT1), ATM/Nsb complex (SIRT1), and Poly (ADP-ribose) polymerase (SIRT6) may indicate a beneficial antiretroviral effect of sirtuin inhibitors as well as their beneficial influence on the post-integration repair process necessary for the incorporation of the viral DNA into the host genome, especially in the case of treatment with INSTIs [35]. Further research is required to confirm the existence of such a relationship.

However, it should be taken into account that sirtuins may participate in many other antagonistic signaling pathways, and therefore the role of sirtuins in the course of HIV cannot be clearly assessed. PIs have a significant impact on the metabolic process, which is one of the causes of side effects during cART [36]. The authors’ study showed that PIs (lopinavir and ritonavir or darunavir and cobicistat) significantly reduced the expression of SIRT1.

So far, little data is available on the involvement of SIRT3 in viral infections. Single data refer to the effect of SIRT3 on Hepatitis B Virus (HBV) progression. Ren et al. showed that silencing the SIRT3 gene enhanced HBV transcription and replication in primarily human hepatocytes and HepG2 cells. The authors point out that the repression of HBV transcription by SIRT3 is related to the decreased binding of host RNA polymerase II and Yin Yang transcription factor 1 (YY1) to covalently closed circular DNA (cccDNA) of HBV. SIRT3 is also considered a limiting factor for oxidative stress caused by HBV X protein (HBx), and thereby it also limits the replication of the virus [37]. SIRT3 influences antioxidant processes through deacetylation and increasing the activity of the antioxidant enzymes–SOD2 and CAT [26].

Yu et al. observed increased reactive oxygen species (ROS) production in CD8+ and CD4+ T cells in the course of HIV infection, as well as changes in mitochondrial membrane potential and mitochondrial mass compared to cells isolated from HIV–uninfected individuals [38]. The above data may indicate a potential role of SIRT3 in the course of HIV infection through its antioxidant properties and its regulation of mitochondrial homeostasis. In the authors’ own study, no significant differences were found in the level of SIRT3 in the plasma of HIV-infected men compared to the control group. The cellular localization of SIRT3 in mitochondria may prevent the ability to assess its changes in the course of HIV infection. More research is needed to confirm the obtained results.

Among the three examined sirtuins, the greatest changes were found in SIRT6 expression in the plasma of HIV-infected men. The data obtained may suggest a beneficial effect of antiretroviral therapy on the level of SIRT6 in HIV positive patients. However, no significant differences between types of therapy were found.

The authors’ own study demonstrated a significantly lower SIRT6 level in HIV-infected men compared to the control group. The exact mechanism explaining the influence of SIRT6 on the course of HIV is still unknown. One of the suggested mechanisms is the activation of SIRT6 by Interferon type 1 (IFN-1) signaling as a result of HIV infection. Hardy et al. showed that IFN-1 activity was significantly higher in HIV-infected patients compared to healthy controls. Moreover, plasma levels of IFN-1 were inversely correlated with CD4+ cell count and positively correlated with HIV RNA [39].

The obtained differences in the level of SIRT6 before and after treatment may also be related to the improvement of parameters such as: LT CD4+, CD8+ count, HIV RNA and the patients’ improved clinical condition as a result of antiretroviral therapy. Such a relationship was observed in the case of SIRT6 and LT CD8+ count.

A significant increase in the level of SIRT6 was associated with an increase in LT CD8+ count after one year of antiretroviral therapy, demonstrating the beneficial effect of cART not only on the increase of SIRT6 expression but also on the response of the immune system. HIV-specific CD8+ T cells are prone to apoptosis, which may affect their ability to control HIV infection. CD8+ lymphocyte-mediated immune responses play a key role in controlling infection, increasing the survival and effector function of HIV-specific CD8+ T cells as well as their ability to control HIV [40].

## 4. Materials and Methods

### 4.1. Patient Characteristics

The study group consisted of 53 HIV-infected men with a mean age of 34 years who were patients at the Center for Preventive and Therapeutic Infectious Diseases and Addiction Therapy in Wroclaw as well as in the Department of Infectious Diseases, Liver Diseases and Acquired Immune Deficiencies of the Medical University of Wroclaw. The control group consisted of 35 healthy HIV-negative males with a mean age of 36 years and without any chronic or inflammatory diseases such as diabetes mellitus, cardiovascular diseases or hepatitis B or C virus infection. In the group of HIV-infected patients, inclusion criteria were the patient′s consent for tests, confirmation of the presence of HIV infection, and cART use. Exclusion criteria were diseases such as diabetes, cancer, hypertension, neurocognitive diseases, and especially urinary tract diseases as well as concomitant use of drugs other than cART.

In the case of the HIV-infected men, blood was drawn twice: before and one year after antiretroviral therapy. Whole human blood (5 mL) was collected from both groups (patients and control) in a fasting state. Blood samples were taken into EDTA-treated tubes (Sarstedt, Warsaw, Poland). Tubes were centrifuged by MPW-350 laboratory centrifuge (MPW Instruments, Poland) at 1500× *g* for 10 min to separate the plasma. Plasma was removed and placed in Eppendorf tubes and stored at −80 °C for further investigation.

HIV-infected men were treated with two therapeutic regimens, which included two NRTIs (emtricitabine and tenofovir alafenamide) in combination with PIs (ritonavir-boosted lopinavir or cobicistat-boosted darunavir) or INSTIs (dolutegravir).

Data on lymphocytes T CD4+ and CD8+ count, HIV viral load and biochemical parameters such as TC, LDL, HDL, TG, FBG and BMI were obtained from medical records.

### 4.2. Determination of SIRT1, SIRT3, SIRT6 Levels in Plasma of HIV-Infected Men and Healthy Controls

The measurement of sirtuins concentrations was performed by the enzyme-linked immunoassay (ELISA) method using Human Sirtuin 1 ELISA Kit (Cat.No E2557Hu), Human Sirtuin 3 ELISA Kit (Cat.No E2559Hu), and Human Sirtuin 6 ELISA Kit (Cat.No E2562Hu) Bioassay Technology Laboratory (BT Lab; Shanghai Korain Biotech Co Ltd., Shanghai, China) according to the manufacturers’ instructions. Standards and serum samples were added into a 96-well plate. After adding the biotin-conjugated anti-SIRT1/SIRT3/SIRT6 antibody and streptavidin-horseradish peroxidase, the plate was incubated for 60 min at 37 °C. The wells were then washed five times with wash buffer. Substrate solutions A and B were added, and the plate was incubated for 10 min at 37 °C for color development. Finally, the reaction was stopped by the stop solution. The intensity of color in each well was measured at 450 nm with a microplate reader (STAT FAX 2100, Palm City, FL, USA).

### 4.3. Statistical Analysis

Statistical analysis was performed using the Statistica 13.3 PL program (StatSoft, Cracow, Poland). For measurable variables, medians and the range of variability (minimum and maximum values) were calculated. For qualitative variables, the frequency of their occurrence (percentage) was calculated. All investigated quantitative variables were checked with the Shapiro-Wilk test to establish the type of distribution. Variables with abnormal distribution were presented as the median and interquartile range (IQR) 25-75%. Results with normal distribution were presented as the mean ± standard deviation (SD). The comparison of qualitative variables between the groups was made using the chi-square test (χ^2^). Since the obtained results did not have the characteristics of a normal distribution, non-parametric tests were used. The Mann–Whitney U test was used for two independent samples (HIV-infected men, control). For dependent samples (HIV-infected men before and after cART), the non-parametric Wilcoxon test was used. The Kruskal–Wallis test was used to compare multiple independent samples. The obtained results were also analyzed in patient subgroups, divided according to: CD4+ count (below and above 300 cells/µL), CD8+ count (below and above 1000 cells/µL), HIV RNA (below and above 100,000 copies/mL) and the type of therapeutic regimen (INSTIs or PIs). For all analyses, *p* < 0.05 was accepted as a significant value.

## 5. Conclusions

This is the first study of the expression of SIRT1, SIRT3 and SIRT6 in HIV-infected men. It is also the first study using clinical material (plasma). The levels of all examined sirtuins were reduced in the plasma of HIV-infected men compared to non-HIV infected men, suggesting a negative effect of HIV infection on their expression in one year of observation. There was no significant effect on the level of SIRT1 and SIRT3 after the implementation of one year of antiretroviral therapy, which depended on HIV viral load and the CD4+ and CD8+ T lymphocytes count. The greatest changes in expression were demonstrated in the case of SIRT6, the levels of which increased significantly after the use of cART, thus proving the beneficial effect of the implemented antiretroviral therapy on the level of this enzyme as well as the therapy’s relationship with HIV viral load, LT CD8+ count, and plasma levels of SIRT6, which may, in turn, reflect the body’s immune response to HIV infection. Interesting results were obtained by analyzing the expression of sirtuins depending on the cART treatment regimen. The expression of all examined sirtuins (SIRT1, SIRT3, and SIRT6) was higher after cART with INSTIs; in the case of SIRT1, the difference was statistically significant. Protease inhibitors (PIs) significantly lowered the level of all sirtuins, which may indicate a significant role of sirtuins in response to antiretroviral therapy. However, the mechanism of these processes is still unknown.

### Future Perspectives

The obtained data suggest the influence of SIRT6 and SIRT1 in the course of HIV infection and cART therapy. The obtained results indicate a need for further research on sirtuin expression, which could potentially create new perspectives in the treatment or optimization of therapy as well as enable better monitoring of the course of HIV infection. The provided explanation of changes in sirtuin-regulated pathways will enable a more detailed understanding of their importance for the treatment of HIV patients in the future. Although the regulation of sirtuin activity can be identified as a potential target of therapy, the above data indicate the need for further research on the role of sirtuins in the course of HIV infection, especially of SIRT6, for which the greatest changes were observed. The exact molecular mechanisms involved are yet to be understood, and a specification of the viral or host genes regulated by SIRT6, or vice versa, is still required.

## Figures and Tables

**Table 1:** Demographic and biochemical data of HIV-infected patients before (**A**) and after cART (**B**) and control group (**C**) with statistical analysis.

Groups Characteristic	A (*n* = 53)	B (*n* = 53)	C (*n* = 34)	*p* *
	Me (IQR)	Me (IQR)	Me (IQR)	
Age (Y)	33 (28–40)	34 (29–41)	36 (30–43)	NS
BMI [kg/m2]	24.15 (21.55–24.80)	24.00 (21.56–24.81)	22.30 (18.00–26.80)	NS
FBG [mg/dL]	96.00 (91.90–98.10)	98.90 (92.40–103.90)	95.00 (87.20–99.20)	NS
TC [mg/dL]	174.90 (156.00–187.00)	174.00 (155.00–189.00)	180.00 (165.00–195.00)	NS
LDL [mg/dL]	96.00 (85.00–103.00)	99.06 (80.00–110.00)	100.00 (96.00–115.00)	NS
HDL [mg/dL]	76.60 (63.00–84.00)	77.15 (67.00–82.00)	75.00 (59.00–101.00)	NS
TG [mg/dL]	149.00 (119.00–160.00)	157.20 (100.00–162.00)	163.00 (151.00–175.00)	NS

Abbreviations: cART–combined antiretroviral therapy; A–HIV-infected patients before cART; B–HIV-infected patients after cART; C–control group; BMI–Body Mass Index; FBG–fasting blood glucose; TC–total cholesterol; LDL–low-density lipoprotein; HDL–high-density lipoprotein; TG–triglycerides; Me–median; IQR–interquartile range; N–number of participants; NS–not statistically significant; * Kruskal–Wallis test.

**Table 2:** Immunological data of HIV-infected men groups before (**A**) and after cART (**B**) with statistical analysis.

Groups Characteristic	A	B	*p* *
	Me (IQR)	Me (IQR)	
HIV RNA [copies/mL]	148,000 (5190–245,000)	20 (15–34)	<0.001
CD4+ cell count [cells/µL]	340 (234–386)	570 (398–762)	<0.001
CD8+ cell count [cells µL]	999 (717–1190)	855 (706–1062)	0.004

Abbreviations: cART–combined antiretroviral therapy; A–HIV-infected patients before cART; B–HIV-infected patients after cART; C–control group; Me–median; IQR–interquartile range; NS–not statistically significant; * Wilcoxon test.

**Table 3:** Results for SIRT1, SIRT3 and SIRT6 in the plasma of HIV-infected men before (**A**) and after cART (**B**) and in the control group (**C**) with statistical analysis.

Groups	A	B	C	*p* *	post-hoc
	Me (IQR)	Me (IQR)	Me (IQR)		
SIRT1 [ng/mL]	7.20 (4.00–21.70)	4.70 (2.20–64.00)	8.50 (2.70–24.10)	0.305	A:C = NS B:C = NS A:B = NS
SIRT3 [ng/mL]	5.80 (4.00–21.10)	5.70 (2.00–29.10)	8.00 (2.70–21.10)	0.131	A:C = NS B:C = NS A:B = NS
SIRT6 [ng/mL]	2.80 (0.80–10.50)	4.40 (2.20–23.20)	7.30 (2.30–19.30)	0.003	A:C = 0.007 B:C = NS A:B = 0.022

Abbreviations: cART–combined antiretroviral therapy; SIRT1, SIRT3, SIRT6–sirtuin 1, 3, 6, respectively; A–HIV-infected patients before cART; B–HIV-infected patients after cART; C–control group; Me–median; IQR–interquartile range; NS–not statistically significant; * Kruskal–Wallis test.

**Table 4:** Results for SIRT1, SIRT3 and SIRT6 in the plasma of HIV-infected men before cART (**A**) and after cART (**B**) subgrouped according to LT CD4+ count.

	CD4+ Count ≤ 300 [Cells/µL]		*p* *
	Me (IQR)	Me (IQR)	
	A	B	
SIRT1 [ng/mL]	12.00 (4.10–24.80)	6.90 (2.50–19.80)	NS
SIRT3 [ng/mL]	15.45 (4.50–42.00)	6.80 (1.90–61.50)	NS
SIRT6 [ng/mL]	3.80 (1.50–25.50)	5.80 (1.90–79.00)	NS
	**CD4+ count > 300 [cells/µL]**		
	**Me** **(IQR)**	**Me** **(IQR)**	
SIRT1 [ng/mL]	5.70 (3.60–16.60)	4.70 (2.20–72.20)	NS
SIRT3 [ng/mL]	5.30 (3.50–11.40)	5.70 (2.00–47.80)	NS
SIRT6 [ng/mL]	1.80 (0.70–8.30)	4.40 (2.10–37.70)	NS

Abbreviations: cART–combined antiretroviral therapy; SIRT1, SIRT3, SIRT6–sirtuin 1, 3, 6, respectively; A–HIV-infected patients before cART; B–HIV-infected patients after cART; Me–median; IQR–Interquartile range; NS–not statistically significant; * Wilcoxon test.

**Table 5:** Results for SIRT1, SIRT3 and SIRT6 in the plasma of HIV-infected men before cART (**A**) and after cART (**B**) in the subgroup with LT CD8+ count ≤ 1000 cells/µL and LT CD8+ and in the subgroup with LT CD8+ count >1000 cells/µL with statistical analysis.

	CD8+ Count ≤ 1000 [Cells/µL]		*p* *
	Me (IQR)	Me (IQR)	
	A	B	
SIRT1 [ng/mL]	10.50 (4.30–24.80)	5.90 (2.20–68.55)	NS
SIRT3 [ng/mL]	9.10 (4.40–42.00)	6.45 (2.05–43.65)	NS
SIRT6 [ng/mL]	3.55 (1.20–25.50)	6.00 (2.20–37.20)	0.04
	**CD8+ count > 1000 [cells/µL]**		
	**Me** **(IQR)**	**Me** **(IQR)**	
SIRT1 [ng/mL]	4.60 (3.60–16.60)	3.60 (2.20–22.60)	NS
SIRT3 [ng/mL]	4.80 (3.40–11.40)	2.50 (1.90–15.80)	NS
SIRT6 [ng/mL]	1.65 (0.60–5.90)	2.80 (2.10–16.50)	0.01

Abbreviations: cART–combined antiretroviral therapy; SIRT1, SIRT3, SIRT6–sirtuin 1, 3, 6, respectively; A–HIV-infected patients before cART; B–HIV-infected patients after cART; Me–median; IQR–Interquartile range; N–number of participants; NS–not statistically significant; * Wilcoxon test.

**Table 6:** Results for SIRT1, SIRT3 and SIRT6 in the plasma of HIV-infected men before cART (**A**) in the subgroup with HIV RNA ≤ 100,000 copies/mL, and in the subgroup with HIV RNA > 100,000 copies/mL.

Group A	HIV RNA ≤ 100,000 [Copies/mL] (*n* = 22)	HIV RNA > 100,000 [Copies/mL] (*n* = 31)	*p* *
	Me (IQR)	Me (IQR)	
SIRT1 [ng/mL]	5.25 (2.90–16.30)	8.90 (4.00–68.90)	NS
SIRT3 [ng/mL]	5.15 (4.10–16.20)	7.80 (3.60–48.90)	NS
SIRT6 [ng/mL]	1.80 (0.60–5.90)	3.60 (0.90–25.50)	NS

Abbreviations: cART–combined antiretroviral therapy; SIRT1, SIRT3, SIRT6–sirtuin 1, 3, 6, respectively; group A–HIV-infected patients before cART; Me–median; N–number of participants; IQR–Interquartile range; NS–not statistically significant; * Mann–Whitney *U* test.

**Table 7:** Results for SIRT1, SIRT3 and SIRT6 in the plasma of HIV-infected men after cART (**B**) in the subgroup treated with Protease inhibitors (PIs) and the subgroup treated with Integrase transfer inhibitors (INSTIs) with statistical analysis.

Group B	Pis (*n* = 25)	INSTIs (*n* = 26)	*p* *
	Me (IQR)	Me (IQR)	
SIRT1 [ng/mL]	3.05 (2.10–19.80)	12.40 (1.40–79.40)	0.025
SIRT3 [ng/mL]	2.95 (1.90–15.80)	9.70 (2.10–51.60)	NS
SIRT6 [ng/mL]	3.20 (2.20–13.70)	10.90 (2.20–37.70)	NS

Abbreviations: cART–combined antiretroviral therapy; SIRT1, SIRT3, SIRT6–sirtuin 1, 3, 6, respectively; B–HIV-infected patients after cART; a–subgroup treated with Protease inhibitors (PIs); b–subgroup treated with Integrase transfer inhibitors (INSTIs); Me–median; IQR–Interquartile range; N–number of participants; NS–not statistically significant; * Mann–Whitney *U* test.

## Data Availability

Not applicable.

## Supplementary Materials

The following supporting information can be downloaded at online, Table S1: Results for SIRT1, SIRT3 and SIRT6 in the plasma of HIV-infected men before cART (A) and after cART (B) subgrouped according to LT CD4+ count.; Table S2: Results for SIRT1, SIRT3 and SIRT6 in the plasma of HIV-infected men before cART (A) and after cART (B) in the subgroup with LT CD8+ count ≤ 1000 cells/µL and LT CD8+ and in the subgroup with LT CD8+ count > 1000 cells/µL with statistical analysis.
