# Supplementary material for: The Effect of Antiretroviral Therapy on SIRT1, SIRT3 and SIRT6 Expression in HIV-Infected Patients
Source: Molecules. 2022 Feb 17;27(4):1358. doi: 10.3390/molecules27041358 (PMC8879865; doi:10.3390/molecules27041358)
Supplement: Supplementary file 1 [file molecules-27-01358-s001.zip › molecules-1575770-supplementary.pdf]

Table S1. Results for SIRT1, SIRT3 and SIRT6 in the plasma of HIV–infected men before cART (**A**) and after cART (**B**) subgrouped according to LT CD4+ count.

**Group A**	**CD4+ count ≤ 300 [cells/μL] (n = 18) (a)**	**CD4+ count > 300 [cells/μL] (n = 35) (b)**	*p* *
	**Me (IQR)**	**Me (IQR)**	
SIRT1 [ng/mL]	12.00 (4.10-24.80)	5.70 (3.60-16.60)	NS
SIRT3 [ng/mL]	15.45 (4.50-42.00)	5.30 (3.50-11.40)	NS
SIRT6 [ng/mL]	3.80 (1.50-25.50)	1.80 (0.70-8.30)	NS
**Group B**	**CD4+ count ≤ 300 [cells/μL] (n = 10)**	**CD4+ count > 300 [cells/μL] (n = 43)**	*p* *
	**Me (IQR)**	**Me (IQR)**	
SIRT1 [ng/mL]	6.90 (2.50-19.80)	4.70 (2.20-72.20)	NS
SIRT3 [ng/mL]	6.80 (1.90-61.50)	5.70 (2.00-47.80)	NS
SIRT6 [ng/mL]	5.80 (1.90-79.00)	4.40 (2.10-37.70)	NS

Abbreviation: cART–combined antiretroviral therapy; SIRT1, SIRT3, SIRT6–sirtuin 1,–3,–6, respectively; A–HIV–infected men before cART; B–HIV–infected men after cART; a–subgroup with CD4+ count ≤ 300 cells/μL; b–subgroup with CD4+ count > 300 cells/μL; Me–median; IQR–Interquartile range; N–number of participants; NS–not statistically significant; * U Mann Whitney test.

Table S2. Results for SIRT1, SIRT3 and SIRT6 in the plasma of HIV–infected men before cART (**A**) and after cART (**B**) in the subgroup with LT CD8+ count ≤1000 cells/μL and LT CD8+ and in the subgroup with LT CD8+ count >1000 cells/μL with statistical analysis.

**Group A**	**CD8+ count ≤ 1000 [cells/μL] (a) (n = 26)**	**CD8+ count > 1000 [cells/μL] (b) (n = 27)**	p *
	**Me (IQR)**	**Me (IQR)**	
SIRT1 [ng/mL]	10.50 (4.30-24.80)	4.60 (3.60-16.60)	NS
SIRT3 [ng/mL]	9.10 (4.40-42.00)	4.80 (3.40-11.40)	NS
SIRT6 [ng/mL]	3.55 (1.20-25.50)	1.65 (0.60-5.90)	NS
**Group B**	**CD8+ count ≤ 1000 [cells/μL] (a) (n = 36)**	**CD8+ count > 1000 [cells/μL] (b) (n = 17)**	p *
	**Me (IQR)**	**Me (IQR)**	
SIRT1 [ng/mL]	5.90 (2.20-68.55)	3.60 (2.20-22.60)	NS
SIRT3 [ng/mL]	6.45 (2.05-43.65)	2.50 (1.90-15.80)	NS
SIRT6 [ng/mL]	6.00 (2.20-37.20)	2.80 (2.10-16.50)	NS

Abbreviation: cART–combined antiretroviral therapy; SIRT1, SIRT3, SIRT6–sirtuin 1,–3,–6, respectively; A–HIV-infected patients before cART; B–HIV-infected patients after cART; a–subgroup with CD8+ count ≤ 1000 (cells/μL); b–subgroup with CD8+ count > 1000 (cells/μL); C–control group (values presented in Table 2); Me - median; IQR–Interquartile range; N–number of participants; NS–not statistically significant; * U Mann Whitney test.
